# Microstructure and Electro-Physical Properties of Sn-3.0Ag-0.5Cu Nanocomposite Solder Reinforced with Ni Nanoparticles in the Melting-Solidification Temperature Range

**DOI:** 10.1007/s11669-017-0532-0

**Published:** 2017-03-10

**Authors:** A. Yakymovych, Yu. Plevachuk, V. Sklyarchuk, B. Sokoliuk, T. Galya, H. Ipser

**Affiliations:** 1 Department of Inorganic Chemistry – Functional Materials, University of Vienna, Althanstraße 14, 1090 Vienna, Austria; 2 Department of Metal Physics, Ivan Franko National University of Lviv, Kyrylo i Mephodiy str. 8, Lviv, 79005 Ukraine; 3 School of Applied Science, Mongolian University of Sciences and Technology, 8th Khoroo, Baga toiruu 46/520, Ulaanbaatar, 14191 Mongolia

**Keywords:** electrical conductivity, microstructure, Ni nanoparticles, Sn-3.0Ag-0.5Ag, x-ray analysis

## Abstract

The electrical conductivity of nanocomposite Sn-3.0Ag-0.5Cu alloys with two different weight percentages of Ni nanoparticles (1.0 and 2.0 wt.%) was measured over a wide temperature range. The samples were produced using a cold pressing method: Sn-3.0Ag-0.5Cu powder and Ni nanopowder were mechanically mixed and pressed into 8 mm diameter rods. Ni nanoparticles were synthesized via a chemical reduction method and characterized by a core/shell structure. Temperature dependencies of the electrical conductivity revealed a hysteresis between the heating and cooling curves in a wide temperature range above the melting temperature. This fact is connected with structure transformations accompanied by a dissolution of Ni nanoparticles, which should be retarded due to an oxide/hydroxide shell on the surface of the nanoparticles. A microstructure analysis of the samples in the solid state showed a fine distribution of intermetallic compounds in the Sn-based matrix. The Ni atoms substituted for Cu atoms in the Cu_6_Sn_5_ compound forming a (Cu,Ni)_6_Sn_5_ phase.

## Introduction

The increasing scientific interest for Sn-based nanocomposite alloys with small additions of various metals,[1–4] oxides[5–8] and carbon[9–12] in nanosized form relates to the possible application of these materials as an alternative to commercial lead-free solders. As pointed out in two literature reviews,[13,14] improved mechanical properties and a reinforced microstructure of solder joints using nanocomposite Sn-Ag-Cu (SAC) alloys compared to those without nanoinclusions, revealed new possibilities for the development of currently used commercial lead-free solders. In general terms, the main profit of the nanosized additions is related to a suppression of the extensive growth of the Cu_6_Sn_5_ intermetallic compound (IMC) at the solder/Cu interface towards the solder side. This is achieved due to the spreading of nanoparticles over the IMC’s surface, thereby suppressing the growth of Cu_6_Sn_5_ at the interface as well as in the bulk solder. In particular, the adsorbed nanoparticles at the IMC layer interface hinder the diffusion of Sn atoms from the bulk solder towards the interface and thereby suppress the IMC growth.

Compared to the solid state, there is a limited number of papers dealing with experimental investigations of nanocomposite SAC solders in the liquid state after melting as well as in the semi-solid state.[15–19] It was shown that minor metal nanoadditions have an impact on the microstructure of solidified SAC solders, but practically without any significant change of the melting temperatures.[15–17] Calorimetric measurements revealed that additions of Co in bulk and nanosized form to a liquid Sn-3.8Ag-0.7Cu alloy resulted in additional heat effects during melting of the Co nanoparticles compared to those in bulk form.[19] During the soldering process the solid joint is heated up to approx. 523 K, while the melting temperature of the solder is near 490 K. Therefore, metal nanoparticles should be partly or totally dissolved in the liquid SAC matrix. At this point, experimental data of thermophysical and thermodynamic properties as well as of the structure of the liquid nanocomposite SAC solders with additions of metal nanoparticles should provide necessary information for reliable simulations of the soldering processes.

Ni nanoparticles are among the most investigated metal nanoinclusions into lead-free solders due to their significant impact on the microstructure and mechanical properties of solders or solder joints.[18,20–23] However, to the best of our knowledge, there are no literature data related to the structure and thermophysical properties of nanocomposite SAC solders with nano Ni additions in the liquid state after melting. In this work, we carried out investigations of the electrical conductivity of nanocomposite SAC in the liquid state. The samples were prepared from commercial Sn.-3.0Ag-0.5Cu powder and self-synthesized Ni nanopowder, where a chemical reduction method was used to produce the Ni nanoparticles.

## Experimental Methods

### Synthesis of Nickel Nanoparticles

The synthesis of Ni nanoparticles was performed via a chemical reduction method. Typically 0.5 g of nickel chloride (NiCl_2_; AlfaAesar) was dissolved in 60 ml distilled water as the metal precursor under continued magnetic stirring (*mixture A*). Another solution was obtained by mixing 20 ml of hydrazine monohydrate (N_2_H_4_·H_2_O; AlfaAesar) and 1.0 g of sodium hydroxide (NaOH; AlfaAesar) as the reducing agent in 20 ml distilled water (*mixture* *B*). Then 0.02 g polyvinylpyrrolidone (PVP) as surfactant agent was added to *mixture B*. At the end, *mixture B* was added drop-wise into *mixture A* under magnetic stirring until a strong dark color developed.

At an elevated temperature of 338 K, nickel nanoparticles were formed after about 30 min. The chemical reaction that reduced nickel chloride is given by:

1 $$ 2{\text{Ni}}^{2 + } + {\text{N}}_{ 2} {\text{H}}_{ 4} + 4{\text{OH}}^{ - } \to 2{\text{Ni }} + {\text{N}}_{2} + 4{\text{H}}_{ 2} {\text{O}} $$

After the reaction was complete, the obtained precipitates were separated from the remaining solution by centrifugation at 4000 rpm for 30 min, rinsed several times with a large amount of distilled water and absolute ethanol to remove the excess amount of surfactants, filtered, and finally dried in vacuum for one day at room temperature.

### Preparation of Nanocomposite Solders

The composite (SAC305)_100−x_(nanoNi)_x_ alloys were prepared by mixing Sn-3.0Ag-0.5Cu (composition in mass%; from now on SAC305) alloy in powder form (average particle size 31 μm, SAC305 powder, Kester, U.S.A.) with 1.0 and 2.0 wt.% Ni nanoparticles (average size 250 nm). Mechanical dispersion of the nanoparticles in the solder powder was achieved by mechanically mixing at room temperature for approximately 30 min using a Retsch mixer (Retsch MM301). After that the (SAC305)_100−x_(nanoNi)_x_ powders were pressed into pellet form (about 3 mm diameter and 10 mm height). All operations with Ni nanoparticles were performed in a glove box (M.Braun, LabMaster 130).

### Electrical Conductivity Measurements

The electrical conductivity measurements were carried out by the 4-point method in an argon atmosphere. Graphite electrodes for current and potential measurements were placed in the wall of the vertical cylindrical boron nitride ceramic measuring cell along its vertical axis. The potential electrodes were provided with thermocouples for temperature measurements. Single thermoelectrodes of these thermocouples were used for electrical conductivity determination. The melt temperature was determined by WRe-5/20 thermocouples located in close contact with the liquid. Further details of this method and its experimental realization have been described by Plevachuk and Sklyarchuk.[24] Each sample was inserted into the cell directly inside a high-pressure vessel. Thus, the actual sample composition was accurate within a tolerance of 0.02 wt.%. The resultant error of the electrical conductivity measurements is about 2%.

### Materials Characterizations

The crystal structure of the nanoparticles themselves and the phase composition of the samples after electrical conductivity measurements were analyzed on a Bruker D8 diffractometer at ambient temperature. The diffractometer operates in the *θ/2θ* mode using Ni-filtered CuK_α_ radiation. Indexing of the phases was supported by the Inorganic Crystal Structural Database (ICSD). Rietveld refinement of the XRD patterns was done with the Topas3^®^ software provided by Bruker AXS. The morphology of as-synthesized nanoparticles and samples was observed by a scanning electron microscope (SEM) Zeiss Supra 55 VP. The excitation energy of the electron beam was 15-20 kV; backscattered electrons (BSE) were detected in order to visualize the surfaces of the samples. The chemical analyses of the sample phases were performed using the energy dispersive x-ray (EDX) technique with four characteristic spectral lines of Ni, Cu (K–line) and Ag, Sn (L-line).

## Results and Discussion

**Fig. 1:**
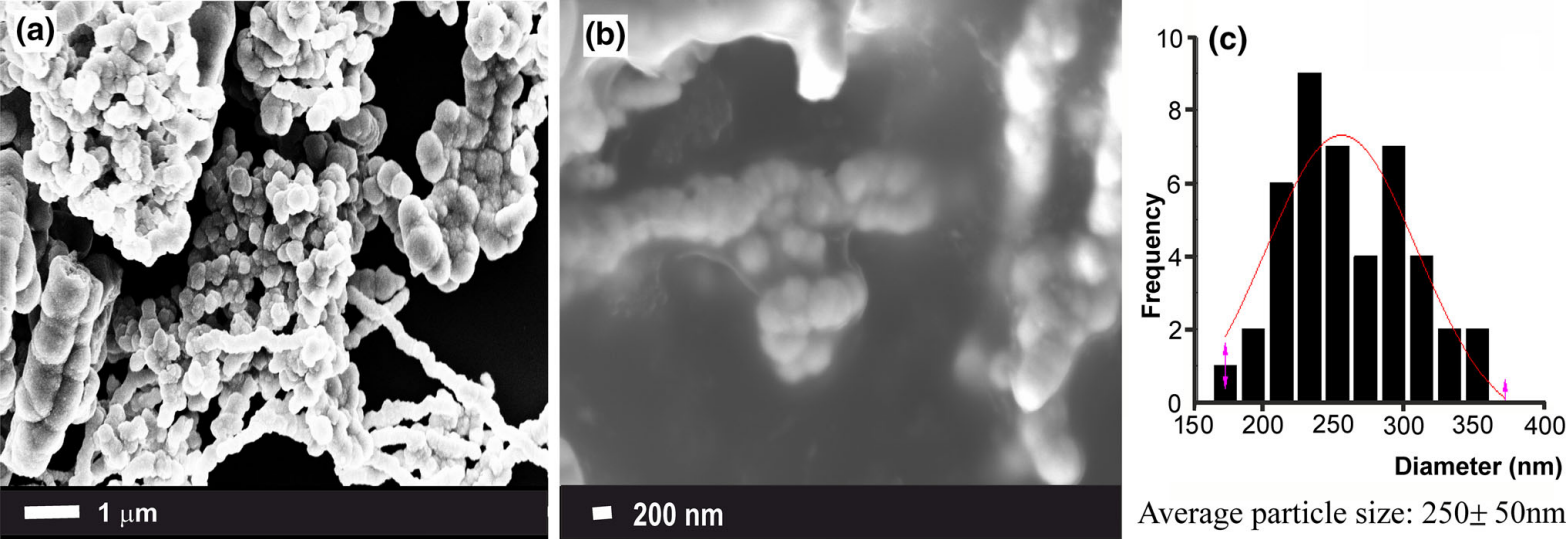
SEM images of as-synthesized Ni nanoparticles (a, b) and distribution of their particle sizes (c)

SEM investigations of the morphology of as-synthesized Ni nanoparticles showed that they formed, rather randomly, large aggregations (Fig. 1a, b). SEM images with different magnification were used to estimate the average particle size of the Ni nanoparticles which was equal to 250 ± 50 nm (Fig. 1c).

**Fig. 2:**
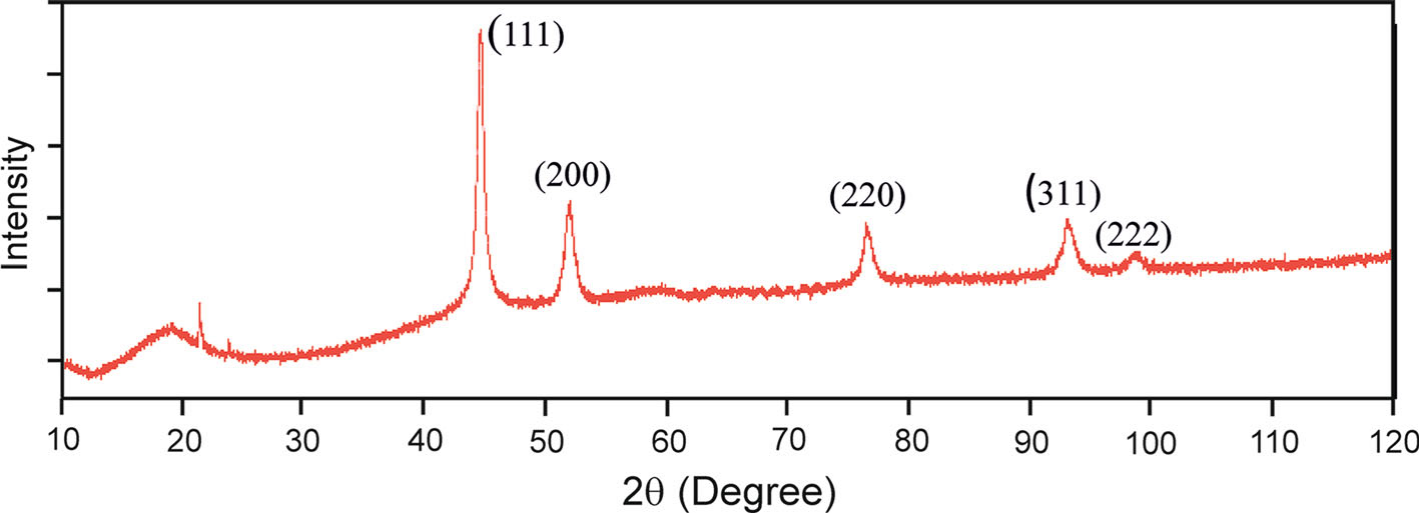
x-ray pattern of as-synthesized Ni nanoparticles

Figure 2 shows an x-ray diffraction pattern of as-synthesized Ni nanopowder which contains the peaks corresponding to the (111), (200), (220) (311) and (222) planes of Ni. According to the performed Rietveld refinement, the Ni nanoparticles have a cubic crystal structure (space group Fm–3 m).

**Fig. 3:**
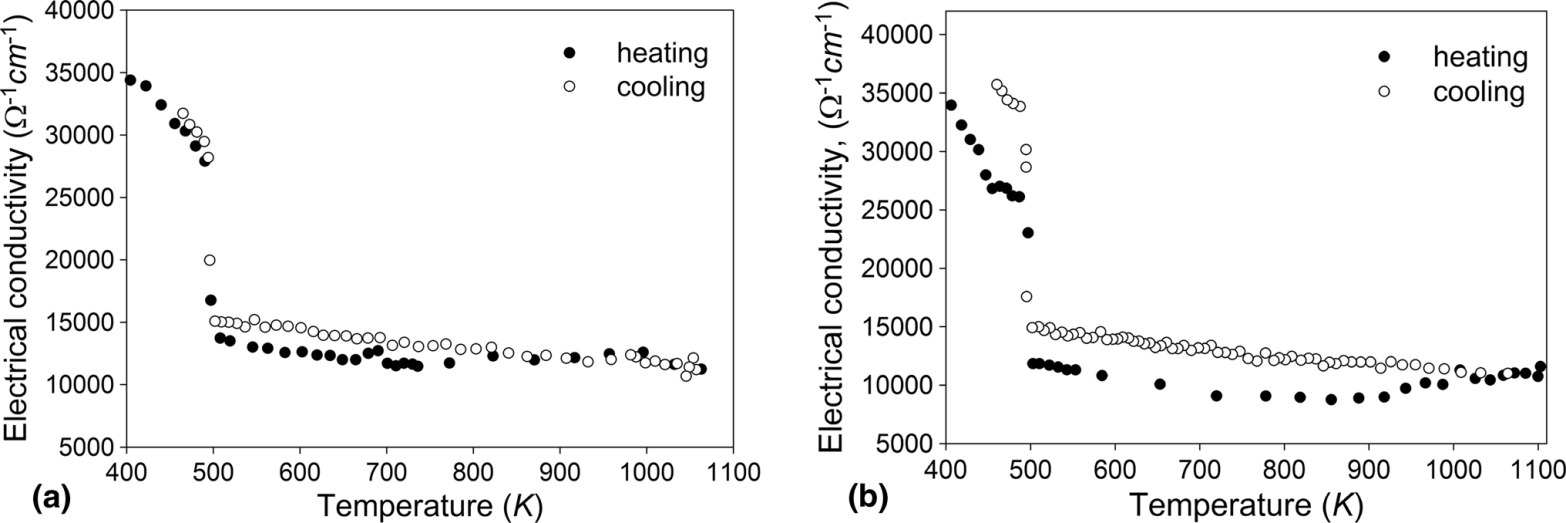
Temperature dependence of the electrical conductivity for the liquid SAC305 + 1 wt.% Ni (a) and SAC305 + 2 wt.% Ni (b) alloys

The experimental temperature dependence of the electrical conductivity during heating and cooling with the same rate of 10 K/min for liquid nanocomposite SAC305 alloys with different contents of Ni nanoparticles in the temperature range between 400 K and 1100 K are presented in Fig. 3. On reaching the melting temperature during heating, the electrical conductivity decreases abruptly from approx. 27,500 Ω^−1^ cm^−1^ to 13,000 Ω^−1^ cm^−1^ for SAC305 + 1 wt.% Ni, and from 27,000 Ω^−1^ cm^−1^ to 12,000 Ω^−1^ cm^−1^ for SAC305 + 2 wt.% Ni. In the liquid state, the electrical conductivity gradually decreases with increasing temperature up to approx. 750 K for liquid SAC305 + 1 wt.% Ni alloy and to approx. 900 K in case of the liquid SAC305 + 2 wt.% Ni alloy.

**Fig. 4:**
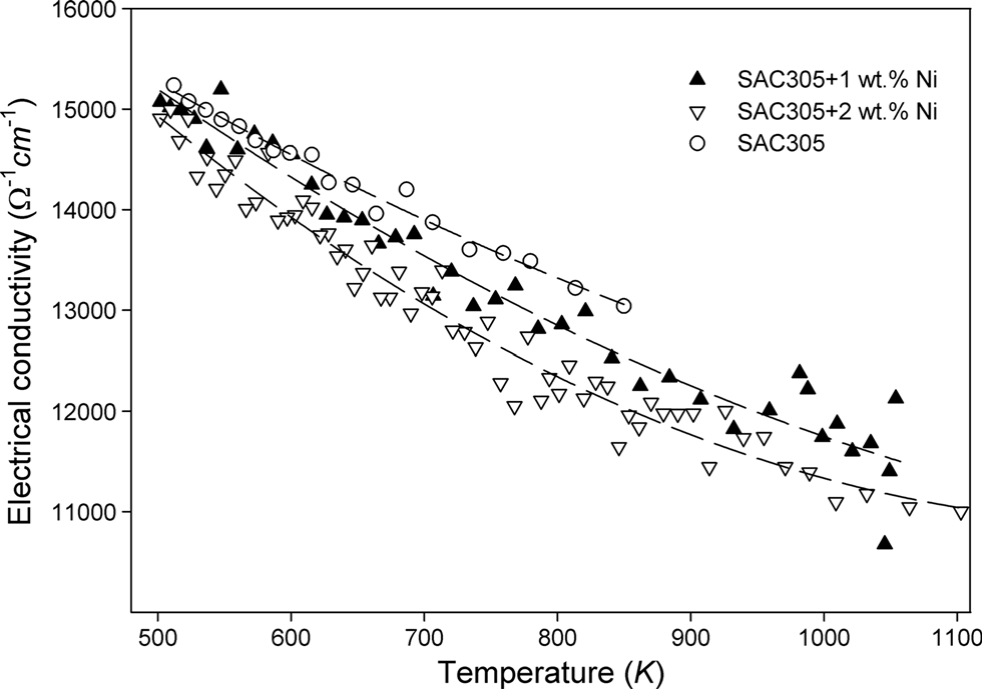
Temperature dependence of the electrical conductivity for the liquid SAC305, SAC305 + 1 wt.% Ni and SAC305 + 2 wt.% Ni alloys on cooling

Further heating is accompanied by a slight increase of the electrical conductivity in both unreinforced SAC305 alloy and SAC305 alloys containing Ni nanoparticles. For the case of the melts containing Ni nanoparticles, this increase could be explained by dissolution of Ni nanoparticles in the SAC305 matrix. The dissolution process was completed at about 800 K in liquid SAC305 + 1 wt.% Ni and 900 K in liquid SAC305 + 2 wt.% Ni. Cooling of the homogeneous melts from 1100 K is accompanied by a smooth increase of the electrical conductivity (Fig. 4).

Fitting the experimental data on cooling when Ni was dissolved completely in liquid solution (Fig. 4) with a parabolic function, the temperature dependence of the electrical conductivity (in Ω^−1^ cm^−1^) can be approximated by the following relationship:

2 $$ \sigma \left( T \right) = 20970{-}13.9T + 0.0046T^{2} $$

for SAC305 + 1 wt.% Ni within the temperature range between about 500 and 1050 K and

3 $$ \sigma \left( T \right) = 22100{-}18T + 0.0072T^{2} $$

for SAC305 + 2 wt.% Ni within the temperature range between about 500 and 1100 K.

The electrical conductivity of the SAC305 alloy is slightly higher than the conductivity of the liquid alloys containing Ni nanoparticles. At the same time, the absolute conductivity values for SAC305 + 1 wt.% Ni are somewhat higher than those for SAC305 + 2 wt.% Ni. It is suggested that the Ni atoms form additional centers of electron scattering, and that an increase of the Ni content leads thus to a decrease of the electrical conductivity.

The microstructure phase composition of the alloys was analyzed after the electrical conductivity measurements using SEM-EDX and x-ray diffraction. The results of the phase analysis along with BSE images of three selected alloys are collected in Table 1. According to the obtained results, no residual pure Ni was found in the samples but the Ni atoms rather substitute for Cu atoms in the Cu_6_Sn_5_ crystals. A similar substitution was reported by Tay et al.[20] who studied the influence of nano Ni additions on the morphology and growth of intermetallic compounds at the interface of SAC387/Cu solder joint.

**Table 1:** SEM-EDX results of samples after electrical resistivity measurements

Sample	Phase 1		Phase 2			Phase 3				Phase 4				SEM image
		Sn at.%		Ag at.%	Sn at.%		Cu at.%	Ni at.%	Sn at.%		Ni at.%	Cu at.%	Sn at.%	
BA1	βSn	100	Ag_3_Sn	70	30	Cu_6_Sn_5_	55	$\cdots$	45					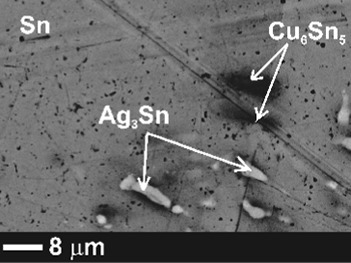
BA2	βSn	100	Ag_3_Sn	75	25	(Cu,Ni)_6_Sn_5_	29-34	15-20	51-56	(Ni,Cu)_3_Sn_4_	31	10	59	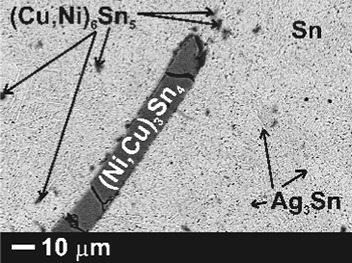
BA3	βSn	100	Ag_3_Sn	75	25	(Cu,Ni)_6_Sn_5_	29-32	18-20	49-53	(Ni,Cu)_3_Sn_4_	28-41	2-13	58-61	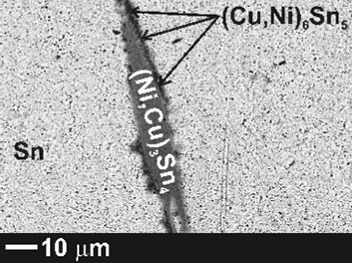

*BA1* bulk SAC305

*BA2* bulk SAC305 + 1 wt.% Ni alloy

*BA3* bulk SAC305 + 2 wt.% Ni alloy

Furthermore, SEM analysis showed that Cu, on the other hand, substitutes for Ni atoms in the intermetallic compound Ni_3_Sn_4_ IMCs. Unlike the finely dispersed small microregions of the (Cu,Ni)_6_Sn_5_ phase, (NiCu)_3_Sn_4_ crystals are present in the form of relative large needles.

## Conclusions

The electrical conductivity of nanocomposite SAC305 alloys without and with two different weight percentages of Ni nanoparticles (1.0 and 2.0 wt.%) was determined. A hysteresis between the heating and cooling curves of the electrical conductivity over a wide temperature range above the melting point is explained by dissolution of Ni nanoparticles in the SAC305 matrix during heating. Cooling of the homogeneous melts from 1100 K is accompanied by a smooth increase of the electrical conductivity.

Higher absolute electrical conductivity values of liquid SAC305 + 1 wt.% Ni as compared to the conductivity of liquid SAC305 + 2 wt.% Ni is due to the fact that the Ni atoms form additional centers of electron scattering, and an increase of the Ni content leads to a decrease of the electrical conductivity.

The microstructure analysis of the samples in the solid state showed a fine distribution of intermetallic compounds in the Sn-based matrix. The Ni atoms substitute the Cu atoms in the Cu_6_Sn_5_ compound forming (Cu,Ni)_6_Sn_5_ crystals.
